# Melanosis of the Subglossian Ganglions Resembling a Gland in Glanders

**Published:** 1896-08

**Authors:** M. Bissange


MELANOSIS OF THE SUBGLOSSIAN GANGLIONS RESEMBLING A GLAND IN GLANDERS. By M. Bissange. A reddish-sorrel draught gelding, about twelve years old, was placed under the care of the writer as not having eaten for several days and coughing very frequently. The animal was in poor condition: his coat was rough, his nostrils red, and he had abundant exudation of a bad nature, purulent, sticky, and of a greenish-yellow color. In the mouth, on the left side, there was a hard indurated gland about as large as a walnut, which was not at all sensitive. It was situated high up, and adhered to the base of the tongue and to the skin. The throat was sensitive, and pinching the larynx caused violent coughing. The pulse was 75, and the respiration agitated and difficult. There was no chancre in the nose. These symptoms led to a diagnosis of serious angina, with the possibility of glanders. During the days which followed, his condition improved, the exudation became clearer, of a more healthy nature, and less abundant. He regained his appetite, but, as the gland did not change, M. Bissange advised the employment of mallein. The owner objected, and he then decided to remove the gland by an operation. The examination of the tumor after it was removed showed that it was merely a tumor of melanotic (mélanique) nature; after the removal of which the horse recovered in a short time. Melanosis of the subglossian ganglions in a sorrel horse, and with the absence of melanotic tumors at the anus, on the genital organs, at the base of the tail, in the parotid region, at the angle of the eye, at the base of the ears, constitutes a very rare clinical fact, which is of a kind to put practitioners on their guard in similar causes which might have serious consequences.—*Ibid.*, October 15, 1895; *Annales de Vét. Méd.*, January and February, 1896.

On to Buffalo!

